# Prediction and Trend Analysis of Regional Industrial Carbon Emission in China: A Study of Nanjing City

**DOI:** 10.3390/ijerph19127165

**Published:** 2022-06-10

**Authors:** Zhicong Zhang, Hao Xie, Jubing Zhang, Xinye Wang, Jiayu Wei, Xibin Quan

**Affiliations:** 1 School of Energy and Mechanical Engineering, Nanjing Normal University, Nanjing 210023, China; huashanzhang74@gmail.com (Z.Z.); jubingzhang@njnu.edu.cn (J.Z.); xinye.wang@njnu.edu.cn (X.W.); wjy412549012@163.com (J.W.); quanxibin2022@163.com (X.Q.); 2 Zhenjiang Institute for Innovation and Development, Nanjing Normal University, Zhenjiang 212016, China

**Keywords:** STIRPAT model, industrial carbon emission, GRPM (1,1) model, influencing factors, scenario analysis

## Abstract

Based on the Stochastic Impacts by Regression on Population, Affluence, and Technology (STIRPAT) model, the impact factors of industrial carbon emission in Nanjing were considered as total population, industrial output value, labor productivity, industrialization rate, energy intensity, research and development (R&D) intensity, and energy structure. Among them, the total population, industrial output value, labor productivity, and industrial energy structure played a role in promoting the increase of industrial carbon emissions in Nanjing, and the degree of influence weakened in turn. For every 1% change in these four factors, carbon emissions increased by 0.52%, 0.49%, 0.17% and 0.12%, respectively. The industrialization rate, R&D intensity, and energy intensity inhibited the increase of industrial carbon emissions, and the inhibiting effect weakened in turn. Every 1% change in these three factors inhibited the increase of industrial carbon emissions in Nanjing by 0.03%, 0.07%, and 0.02%, respectively. Then, taking the relevant data of industrial carbon emissions in Nanjing from 2006 to 2020 as a sample, the gray rolling prediction model with one variable and one first-order equation (GRPM (1,1)) forecast and scenario analysis is used to predict the industrial carbon emission in Nanjing under the influence of the pandemic from 2021 to 2030, and the three development scenarios were established as three levels of high-carbon, benchmark and low-carbon, It was concluded that Nanjing’s industrial carbon emissions in 2030 would be 229.95 million tons under the high-carbon development scenario, 226.92 million tons under the benchmark development scenario, and 220.91 million tons under the low-carbon development scenario. It can not only provide data reference for controlling industrial carbon emissions in the future but also provide policy suggestions and development routes for urban planning decision-makers. Finally, it is hoped that this provides a reference for other cities with similar development as Nanjing.

## 1. Introduction

In recent years, with the rapid development of the economy and the rapid consumption of resources, the excessive emission of greenhouse gases has led to a serious impact on the global climate [1]. As the largest developing country in the world, China is also a major carbon emitter. To shoulder the responsibility of a major country in solving the problem of climate change and promote the rapid development of ecology and civilization. At the 75th UN General Assembly in 2020, President Xi put forward the goal of “striving to peak carbon dioxide emissions by 2030 and strive to achieve carbon neutrality by 2060” [2]. It was pointed out that China should achieve the goal of “double carbon” [3] in the face of climate change.

Carbon emission is not only an environmental problem, but also a development problem. The core of how to reasonably organize the spatial layout under the dual carbon plan lies in how to correctly deal with the balance between regional economy, society, and environment. Scholars have analyzed the relationship between carbon emissions and China’s economic development based on the balanced panel data of 30 provinces in China [4]. Later research analyzed the relationship between carbon emissions and economic development in various provinces and cities in China from the dimensions of space and time [5,6]. It was proven that the environmental Kuznets inverted U-curve was correct, that is, the relationship between economic growth and environmental pollution. In the early stage of the development of a country or region, the degree of environmental pollution increased first and then decreased in an inverted U-shape with the growth of per capita gross domestic product (GDP) [7].

Carbon emissions have become a hot issue in the research of low-carbon economic development at home and abroad. Nowadays, the research on carbon emission by scholars from various countries is mainly divided into two parts: influencing factor analysis and prediction. Factor analysis includes index decomposition analysis (IDA) and structural decomposition analysis (SDA) of carbon emissions. Through the research, it was found that IDA was simple to operate and easy to be used for time series analysis, while LMDI was most widely used [8]. Hasan et al. [9] analyzed the influencing factors of carbon emissions in Bangladesh’s power sector using the LMDI model. Contaminate et al. [10] used the logarithmic mean divisor index (LMDI) method to decompose the sources of changes in the carbon dioxide emission level and intensity of the industry from 2005 to 2017. Chong et al. [11] analyzed the contribution of relevant technology drivers of carbon dioxide emission growth in Malaysia based on the LMDI model. Ehrlich et al. [12] first proposed the IPAT model, pointing out that factors such as population (P), affluence (A), and technical level (T) are the key to environmental pressure (I). Chang et al. [13,14,15] further analyzed the influencing factors between economic growth and the environment by using the IPAT model.

Scholars have continued to dig into the internal relationship of the influencing factors of carbon emissions, expanding the IPAT model to the STIRPAT model, and introduced more parameters into the model. Roberts et al. [16] used the STIRPAT model to find that both population and wealth were important reasons for the increase in carbon dioxide emissions in the southwest the United States. Chikaraishi et al. [17] introduced the urbanization rate and the proportion of tertiary industry into the STIRPAT model and predicted and analyzed the scenarios of different urbanization schemes. It was found that when the per capita GDP and the proportion of tertiary industry were relatively high, it would inhibit the emission of carbon dioxide to a certain extent. Ma et al. [18] combined the GM (1,1) grey model with polynomial regression to predict carbon emissions according to the economic and energy consumption statistics of Jiangsu Province and found that carbon emissions of Jiangsu Province would continue to increase from 2015 to 2020. The growth of per capita gross domestic product GDP has played the most positive role in promoting the growth of carbon emissions. Wu et al. [19], taking Qingdao as an example, the extended STIRPAT model and scenario analysis were introduced to test the impact of different driver combinations on CO_2_ emissions and determine the number and occurrence time of corresponding carbon emissions peaks under different scenarios. Yang et al. [20] established a random impact STIRPAT model by establishing population, impact, and technology regression, and used a non-dominated sorting genetic algorithm II (NSGA-II) to optimize Shanghai’s economic structure adjustment and reduce carbon emissions from the aspects of economic and climate objectives. Yue et al. [21] proposed to consider the impact of economic growth, population growth, energy intensity, and renewable energy shared on carbon emissions in combination with the IPAT model and scenario analysis. The study found that the reduction of energy intensity had a great impact. Zhang et al. [22] analyzed the driving factors affecting China’s CO_2_ and found that the optimization of the industrial structure showed the greatest potential for environmental improvement during 2020–2030 through scenario analysis. Kai et al. [23]—based on the STIRPAT model— used scenario analysis combined with energy transformation and clean coal technology to predict the time and emission trajectory of China’s carbon peak.

In this paper, Nanjing was selected as the research city and the detailed reason was shown in Section 2.4. Zhao et al. [24] discussed the influencing factors of Nanjing’s carbon footprint through the LMDI decomposition model. The results show that the total carbon emission of Nanjing has increased rapidly since 2000, among which the carbon emission from fossil energy utilization was the largest. Economic development, population, and industrial structure were considered as the promoting factors for the increase of Nanjing’s carbon footprint, while the intensity of the industrial carbon footprint was the restraining factor. However, there are few studies on the industrial carbon emissions of Nanjing. 

Sun et al. [25] used the grey model to predict China’s carbon dioxide emissions. It was found that the average absolute percentage error (MAPE) of traditional GM (1,1) was 4.35%, and the MAPE of GM (1,1) optimized by harmony search was 3.93%. Mi et al. [26] used the grey GM model to predict the carbon emission of China’s thermal power enterprises and proposed the optimization of the GM model. They found that the average prediction residual error of the optimized grey model whitening reaction equation decreased from 4.23% to 1.96%. For the traditional GM model, the prediction error is large and the prediction accuracy is low. 

Therefore, in this paper, the gray model was also optimized to improve the prediction accuracy and then was used to predict carbon emissions. A gray rolling prediction GRPM (1,1) [27] model based on the average weakened buffer operator was proposed.

Therefore, this study focused on industrial carbon emissions and took Nanjing as an example. Firstly, the STIRPAT model was used to analyze the main influencing factors of urban industrial carbon emissions and a formula for the influencing factors of carbon emissions was constructed to understand the composition of industrial carbon emissions in Nanjing in more detail. Then, an optimized gray rolling prediction GRPM (1,1) [27] model based on the average weakened buffer operator was proposed for the scenario analysis to predict the industrial carbon emissions in Nanjing from 2021 to 2030 under the influence of the epidemic. The results could help to propose constructive industrial development optimization paths and to point out targeted emission reduction measures to improve more precise guidance for seeking carbon peaking and carbon-neutral paths. The results also provided references for the development of other traditional industrial cities in China or other 35 low-carbon pilot cities.

## 2. Methods, Study Area, and Data Sources

### 2.1. STIRPAT Model

STIRPAT (Stochastic Impacts by Regression on Population, Affluence, and Technology) model is an expanded model of the IPAT model, which further expanded the environmental load (I), population size (P), affluence (A), and technical level (T), and establishes more variables, as follows;

(1) $$I=a\times P^{b}\times A^{c}\times T^{d}\times e$$

where a is the proportional constant term of the model; b, c, and d are the exponential terms, and e is the error term. When a = b = c = d = 1, STIRPAT reverted to the IPAT equation.

Although the current situation of industrial development in Nanjing, the three indicators of population scale, affluence, and technical level in the IPAT model are further expanded into population indicators, wealth indicators, and technical indicators.

#### 2.1.1. Population Indicators

The total population of the whole city is selected as the population index predicted by the model [28]. Industrial development brings more employment opportunities, resulting in a higher population and the increase in the energy consumption and carbon emissions. Therefore, it is of great practical significance to choose the total population as the driving factor of carbon emission prediction.

#### 2.1.2. Wealth Indicators

Industrial output value, labor productivity, and industrialization rate are selected as wealth indicators. Economic development is inseparable from the development of the whole industry. Industrial development also represents the rapid consumption of energy and the rapid increase in carbon emissions. As a new first-tier city, Nanjing’s economic development is also inseparable from the industrial development of the whole city. Industrial output value and industrialization rate represent the importance of local cities to industrial development, while labor productivity reflects the ability of the region to create wealth. Therefore, industrial output value labor productivity and industrialization rate, as the driving factors of model prediction, are representative and typical.

#### 2.1.3. Technical Indicators

The growth of GDP can show the accumulation of wealth, which is the whole phenomenon of society and even a country, and technological progress is the continuous driving force for the continuous improvement of social prosperity and economic development. At the technical level, this paper selects three items: industrial energy intensity, industrial R&D intensity, and energy structure [29]. Industrial energy intensity refers to the energy consumed per unit of industrial output value. Industrial energy intensity is mainly limited by the technical level, especially the instrument and equipment and industrial level. Therefore, the improvement and improvement of technical level play a certain role in reducing the energy consumption per unit of industrial output value and then has an impact on industrial carbon emission. The industrial R&D intensity is the R&D investment and improvement of this instrument and equipment and the industrial level. Industrial R&D intensity indicates the input variable of technical level, while industrial energy intensity indicates the output variable. Energy intensity also indicates the demand for R&D investment for industrial energy types in the whole city. The intensity of industrial R&D is expressed by the intensity of R&D investment, that is, the proportion of internal R&D expenditure in the main business income of the industry, which can indicate the degree of attention paid to the research and development of energy-saving and emission reduction technologies. Industrial R&D funds are mainly invested in the improvement of production technology, especially the scientific research investment in industrial industries with high energy consumption, high pollution, and overcapacity. Improving the production process, improving the technical level, and strengthening energy utilization efficiency are conducive to improving the environment and controlling carbon emissions to a certain extent, as shown in Table 1.

Based on the STIRPAT model coefficient analysis, this paper established and analyzed the industrial carbon emission model of Nanjing as follows.

(2) $$E=a\times P^{b}\times V^{c}\times R^{d}\times L^{e}\times {\mathrm{EI}}^{f}\times {\mathrm{RD}}^{g}\times {\mathrm{ES}}^{h}\times k$$

where E represented the total industrial CO_2_ emissions (10^4^ tons); P is the permanent resident population (10^4^ persons); V refers to the economic level (expressed as Industrial output value, 10^8^ RMB¥); R represented the Labor productivity (expressed as Industrial output value/number of employees yuan/people); L refers to the Industrialization rate (expressed as Ratio of industrial output value to GDP %); EI is the technical level (expressed as energy intensity, i.e., energy consumption per unit GDP, TCE/10^4^ RMB¥); RD is the Industrial R&D intensity (expressed as the percentage of internal expenditure of R&D funds in the main business income of industry %); ES refers to the energy consumption structure (expressed as industrial coal consumption in primary energy consumption %).

### 2.2. Grey Rolling Prediction Model GRPM (1,1)

According to the grey system theory, the latest information in the development trend was usually more able to reflect the characteristics of its future changes than the old information, while the traditional grey prediction modeling only considered the development characteristics of historical data, and rarely brings the new information into the system to consider the future development. Therefore, this topic selected an improved grey model based on the traditional grey model GM (1,1) [30] and the grey rolling prediction model GRPM (1,1), which could fully consider the impact of new information on the development of the system. Because the energy problem is a complex and changeable system, the selection of this model had a certain display significance. Therefore, this paper selected GRPM (1,1) model to predict the industrial carbon emission of Nanjing from 2021 to 2030.

Let the original nonnegative sequence be:

X (0) = [x^(0)^(1), x^(0)^(2), x^(0)^(3), …… x^(0)^(n)] (3)

Processed sequence:

Y (0) = [y^(0)^(1), y^(0)^(2), y^(0)^(3), …… y^(0)^(n)] (4)

where y^(0)^(k) ≥ 0, k = 1, 2, …, n;

Y (1) is the one-time accumulation of sequence y (0):

Y (1) = [y^(1)^(1), y^(1)^(2), y^(1)^(3), …… y^(1)^(n)] (5)

where $y^{\left(1\right)}(k)=\sum_{i=1}^{k}y^{\left(0\right)}\left(i\right)$, k = 1, 2, …, n;

Z (1) is the next generated sequence of sequence y (0):

Z (1) = [z^(1)^(1), z^(1)^(2), z^(1)^(3), …… z^(1)^(n)] (6)

For sequence Z (1), $z^{\left(1\right)}\left(k\right)={\mathrm{ax}}^{\left(1\right)}\left(k\right)+\left(1-a\right)x^{\left(1\right)}\left(k-1\right),\;k=2,\;3$, …, n. Where 0 ≤ a ≤ 1 is the weight. Generally, the mean sequence a = 0.5. The differential equation is established as follows. It is the basic form of the grey rolling prediction model GRPM (1,1).

(7) $$y^{\left(0\right)}\left(k\right)+{\mathrm{az}}^{\left(1\right)}\left(k\right)=\mathrm{kb}+c,\;k=2,\;3$$

Transfer the differential equation to obtain Equation (8). The parameters can be determined by the least square matrix method β. The estimated values of parameters a, b and c are obtained and brought into the equation to obtain the final prediction formula.

(8) $$-{\mathrm{az}}^{\left(1\right)}\left(k\right)+\mathrm{kb}+c=y^{\left(0\right)}\left(k\right),\;k=2,\;3$$

In the grey prediction model, the first point information of the original sequence is usually used as the initial value to replace the prediction data of the first time point. Therefore, the final time corresponding formula of GRPM (1,1) could be sorted as follows.

(9) $$\begin{array}{c} {\hat{y}}^{\left(0\right)}\left(k\right)=\left[\left(\frac{1-0.5a}{1+0.5a}-1\right)y^{\left(0\right)}\left(1\right)+\left(\frac{2b}{1+0.5a}+\frac{c}{1+0.5a}\right)\right]{\left(\frac{1-0.5a}{1+0.5a}\right)}^{k-2}\\ +\sum_{t=0}^{k-3}\left(\frac{b}{1+0.5a}\right){\left(\frac{1-0.5a}{1+0.5a}\right)}^{t} \end{array}$$

where, k = 1, 2, …, n. The above formula can be used to complete the data prediction function.

### 2.3. Scenario Analysis

The scenario analysis method refers to the method of setting different scenarios to realize simulation according to the possible situation of the prediction object on the premise that a phenomenon was coming.

### 2.4. Study Area 

Nanjing is located in the Yangtze River Delta of China. It is one of the regional centers of China’s productivity layout and the main industrial base in China. At the same time, it is also the provincial capital of Jiangsu Province and a national low-carbon pilot city.

The current development situation of Nanjing is from two aspects of industrial structure and industrial energy consumption. Nanjing’s industrial structure adjustment is relatively slow, the proportion of the industry is high, and the industrial output value accounts for more than 30% of Nanjing’s GDP. The total industrial output value of the four major industries of automobile, steel, electronic information manufacturing, and petrochemical new materials in Nanjing only accounts for about 35%, but its comprehensive energy consumption accounts for more than 95%. In particular, the steel, petrochemical, and electric power industries have become large comprehensive emitters; although Nanjing has continued to promote the upgrading of the heavy chemical industry and the innovation of production technology and process in recent years, it is also facing the constraints of investment, cost, and market, and there is great pressure on energy conservation and emission reduction in traditional industries. Second, the proportion of high-energy consuming industries is too high, and the energy consumption per unit output value is low. The six high-energy consuming industries, including chemical raw materials and chemical products manufacturing industry, waste metal and mineral products industry, ferrous metal smelting and rolling processing industry, nonferrous metal smelting and rolling processing industry, petroleum processing, and smelting and nuclear fuel processing industry, and power and thermal power production and supply industry, account for more than 90% of the total energy consumption, and the corresponding output value accounts for only about 30%. From the perspective of energy structure, Nanjing has insufficient diversity of energy structure and high dependence on traditional energy. The traditional energy dominated by raw coal and oil decreased from 81.7% in 2000 to 72.6% in 2016, which is still at a high level.

Taking Nanjing as a case study, this paper selects the optimal industrial development path and puts forward targeted emission reduction measures. It can provide a reference for the development of other traditional industrial cities or low-carbon pilot cities [31].

### 2.5. Data Sources

At present, the carbon emission data of various industries or departments are not directly given in the official data of national or urban statistics, so it needs to be calculated through energy consumption. Select the industrial energy consumption data of Nanjing from 2006 to 2020, and calculate the industrial carbon emission of Nanjing through the IPCC carbon emission accounting method. The basic calculation data in this paper came from the data in the energy purchase, consumption, and inventory table of Industrial Enterprises above Designated Size in Nanjing Statistical Yearbook (2006–2020). According to the data in Appendix A Table A3, the type of terminal energy consumption was divided into eight types: raw coal, coke, crude oil, gasoline, kerosene, diesel, fuel oil, and natural gas. Since the energy consumption in the table is based on the physical quantity, it is necessary to convert these energies into standard coal when calculating the carbon emission. In this paper, the estimation method of regional carbon emission proposed by previous scholars is adopted, and the calculation formula of carbon emission is as follows:

(10) $$C_{rt}=\frac{44}{12}\overset{8}{\underset{i=1}{\sum }}E_{irt}T_{i}F_{i}$$

where 44/12 is the molecular weight of carbon dioxide; $C_{rt}$ refers to the carbon emission caused by energy consumption in year t in region r; $E_{irt}$ refers to the consumption of class i energy in year t in region r. In this paper, the consumption of raw coal, coke, crude oil, gasoline, kerosene, diesel, fuel oil, and natural gas is calculated; $T_{i}$ was the conversion coefficient of class i energy standard coal; $F_{i}$ refers to the carbon emission coefficient of class i energy in China. In this paper, the specific data of Nanjing were calculated by using the standard coal conversion coefficient and carbon emission coefficient provided by IPCC (2006).

## 3. Results and Discussion

### 3.1. Overall Analysis

**Figure 1:**
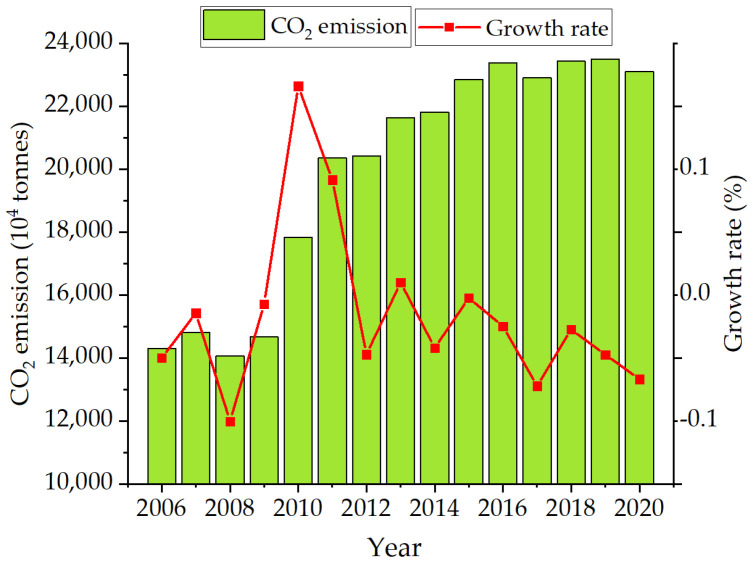
Carbon emission and change trend of Nanjing from 2006 to 2020.

According to Equation (10), this study could calculate the annual carbon emission and the growth rate of Nanjing from 2006 to 2020. The specific calculation results are shown in Figure 1. The carbon emission of Nanjing has maintained an increasing trend since 2006, from 142.95 million tons in 2006 and increasing to 231.07 million tons in 2020, with an overall increase of 62%. During the period from 2006 to 2016, carbon emissions maintained a high-speed growth trend, and during the period from 2016 to 2020, the growth trend of carbon emissions gradually slowed down, or even showed negative growth, indicating that Nanjing had made great achievements in the 13th Five-Year plan, continuously optimizing and upgrading its industrial structure, Shifting the economic focus from the secondary industry manufacturing industry to the tertiary industry service industry.

### 3.2. Fitting Analysis

Before regression fitting, the data imported into SPSS 26.0 software needs to be logarithmicized into the form of a log function, and then Pearson correlation analysis is carried out on the variables of the extended STIRPAT model. The analysis results are shown in Table 2.

Table 1 shows a high correlation between the dependent variables (industrial carbon emission) and their respective variables, and a certain correlation between their respective variables as well. Therefore, there might be some certain multicollinearity between the independent variables.

In Table 3, the adjusted R^2^ was 0.991, close to 1, indicating that the overall regression fitting effect of the equation as well. Next, the variance expansion factor VIF test was carried out on the equation factors. The results show that the variance expansion factor VIF of lnP, lnV, lnR, lnL, lnEI, and lnRD was greater than 10, indicating that there was multicollinearity between them. At this time, to avoid pseudo regression, the least square method or ridge regression method was usually used for analysis. As ridge regression was an improved least square method, which was especially used for collinearity data analysis, this paper selected the ridge regression method to analyze variables. 

**Figure 2:**
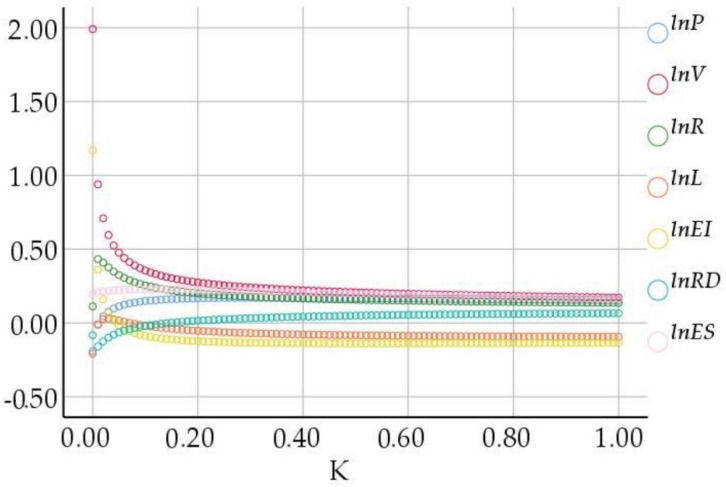
R^2^ diagram corresponding to K value.

**Figure 3:**
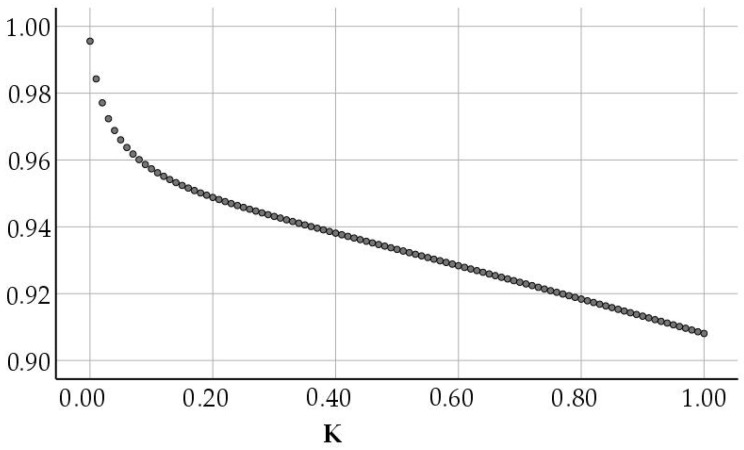
Ridge trace map.

According to the principle that the K value in Figure 2 was as small as possible, R^2^ was as large as possible, and the ridge regression coefficient corresponding to the K value in Figure 3 gradually tended to be stable when the value of K ranges from 0 to 0.09, the ridge regression coefficient corresponding to each variable changed greatly, indicated that its stability was poor. When the value of K reached between 0.10 and 0.20, the ridge regression coefficient corresponding to each variable was basically stable. The determination coefficient was then investigated. When k = 0.10, R^2^ = 0.957 was still large, so the ridge parameter k = 0.10 was taken. When k = 0.1, the regression detection was significant, indicating that the goodness of fit was high and the ridge regression coefficient of each variable tended to be stable, then the standardized ridge regression equation of the model could be obtained as follows;

lnE = 0.4863 lnP + 0.1662 lnV + 0.1164 lnR − 0.0325 lnL − 0.0664 lnEI − 0.0165 lnRD + 0.5184 lnES + 2.1438.(11)

Judging from the standardized ridge regression equation, the four influencing factors of total population, industrial output value, labor productivity, and industrial energy structure were positively correlated with industrial carbon emission in Nanjing. The increase of these four factors would increase industrial carbon emissions. The three influencing factors of industrialization rate, energy intensity, and R&D intensity were negatively correlated with industrial carbon emissions in Nanjing. The increase of these four factors would inhibit the increase of industrial carbon emissions. When the total population, industrial output value, labor productivity, industrial energy structure, industrialization rate, energy intensity, and R&D intensity changed by 1%, it caused changes in energy consumption carbon emissions of 0.49%, 0.17%, 0.12%, 0.52%, −0.03%, −0.07%, and −0.02%, respectively.

### 3.3. Model Data Prediction Analysis

According to the seven factors affecting industrial carbon emissions in Nanjing and the original data on carbon emissions from 2006 to 2020, the GRPM (1,1) model was established. Through the residual test, the average relative error was 3.4% and the accuracy was grade II, which proved that the prediction had high accuracy and was effective.

The model is established according to the industrial carbon emission of Nanjing, considering the addition of new information and constantly updating the sequence to predict the data. The specific steps were as follows:

(1) Set the modeling step of GRPM (1,1) model m = 6, and the initial modeling sequence is as follows.
  $$Y_{1}^{\left(0\right)}=(19,952.68,\;20,356.74,\;20,783.72,\;21,343.95,\;21,951.12,\;22,362.62)$$
(2) For initial sequence $Y_{1}^{\left(0\right)}$, the corresponding grey rolling prediction model was established and parameters were calculated as follows.
  $${\left(a_{1},b_{1},c_{1}\right)}^{T}={\left(-0.0124,252.76,1945.17\right)}^{T}$$
(3) Calculate the first point error of GRPM (1,1). Due to the special prediction mechanism of GRPM (1,1), the latest data or the data predicted for the first time were used as the source of error calculation = 22,935.53, the error was 1.56%.
(4) Add new data and delete old data. Then update the modeling sequence as follows.
  $$Y_{2}^{\left(0\right)}=(20,356.74,\;20,783.72,\;21,343.95,\;21,951.12,\;22,362.62,\;22,584.61)$$

Then repeat (2) to (4) to continuously update the information,

$$\begin{array}{c} Y_{3}^{\left(0\right)}=(20,783.72,\;21,343.95,\;21,951.12,\;22,362.62,\;22,584.61,\;22,854.77)\\ Y_{4}^{\left(0\right)}=(21,343.95,\;21,951.12,\;22,362.62,\;22,584.61,\;22,854.77,\;23,027.17)\\ \ldots \ldots \\ Y_{10}^{\left(0\right)}=(23,228.04,\;23,301.08,\;23,241.34,\;23,348.63,\;23,303.66,\;23,107.12) \end{array}$$

**Figure 4:**
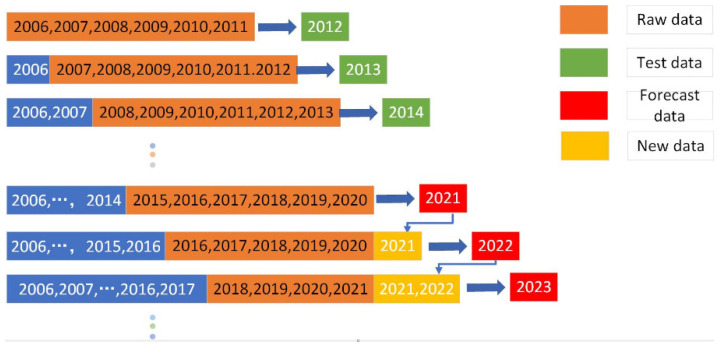
Modeling and prediction process of GRPM (1,1).

Calculate the comprehensive percentage error of the model and complete the modeling process of GRPM (1,1). The modeling flow chart is shown in Figure 4.

The industrial carbon emissions of Nanjing from 2021 to 2030 were predicted by modeling, as shown in Table 4. Through the GRPM (1,1) model, the industrial carbon emission of Nanjing was modeled, and the predicted value of industrial carbon emission of Nanjing from 2021 to 2030 was obtained. Combined with the calculated data, it is found that the highest point of industrial carbon emission in Nanjing appeared in 2019, which was 235.02 million tons, and then gradually decreased.

### 3.4. Carbon Emission Scenario Analysis

This paper made a scenario analysis based on the development of the economy, population, and technology in Nanjing before the outbreak of the epidemic in 2006–2019 and after the outbreak in 2020. Before the outbreak of the epidemic in 2019, economic growth, the continuous rise of entrepreneurial and innovative companies, and the shortage of technical personnel. The city’s industries above the designated size achieved an industrial added value of 287.35 billion yuan, a year-on-year increase of 10.5%, and an average increase of 7.8% in the two years. Among the 36 industry categories in the city, 28 industries achieved year-on-year growth in value-added. However, after the outbreak in 2020, the economy declined by 4% year on year. During the COVID-19 pandemic, to prevent and control the spread of the virus, the government formulated a series of policies and measures, such as extending the Spring Festival holiday, stopping production nationwide, blocking traffic, stopping transportation, isolating employees, and greatly reducing the production of industrial enterprises. At the same time, the prevention and control policies have reduced the number of residents going out, seriously affecting catering, tourism, transportation, film and television, and other related industries. The shutdown, shutdown, and layoffs of enterprises have increased the pressure on young people, increased the cost of childbearing, decreased the willingness to give birth, and the fertility rate has also decreased.

Therefore, after the outbreak of the epidemic, the production of industrial enterprises in the city slowed down and energy consumption decreased. In 2020, the city’s comprehensive energy consumption of industries above the designated size will be 16.11 million tons of standard coal, a year-on-year decrease of 3.5%. Among the 36 industry categories, 31 industries’ energy consumption decreased to varying degrees, with a decline rate of 86.1%. The comprehensive energy consumption of the five high energy-consuming industries of the chemical industry, steel, petroleum processing, electric power, and cement totaled 15.88 million tons of standard coal, a year-on-year decrease of 2.9%, accounting for 96.2% of the city’s industries above designated size. The city’s industries above the designated size consumed a total of 14.75 million tons of coal, a year-on-year decrease of 8.0%, and the consumption decreased by 0.44 million tons compared with the same period. The consumption of coal in cement, petrochemical, steel, and other key nonpower coal industries has been greatly differentiated. The crude oil processing volume of the petrochemical industry decreased by 11.8%, and coal consumption decreased by 4.1%. The iron and steel industry maintained continuous production during the epidemic period, with pig iron output increasing by 12.5% and coal consumption increasing by 10.0% year on year.

Industries related to industrial landmarks, such as artificial intelligence, integrated circuits, and green new energy vehicles, grew against the trend. The industrial added value of the integrated circuit industry increased by 36%, and that of the new energy vehicle industry increased by 18.1%. The output of integrated circuits and industrial robots increased by about 1.5 times and 9 times year-on-year, respectively. The added value of traditional advantageous industries such as computer, communication, and other electronic equipment manufacturing, electrical machinery and equipment manufacturing, railway, shipbuilding, aerospace, and other transportation equipment manufacturing increased by 10.7%, 10.1%, and 8.4%, respectively. 

For the comparison before and after the occurrence of the epidemic, this paper set up the baseline, high-carbon, and low-carbon development scenarios for seven carbon emission factors. First, the trend results of the GRPM (1,1) model combined with the industrial carbon emission data of Nanjing from 2006 to 2020 were used as the baseline scenario, taking into account the current development trend. The second was to continue to be affected by the epidemic in the future, while government departments strengthen the upgrading of highly polluting industrial enterprises to reduce their pollution emissions and achieve green production. This scenario was designed as a low-carbon development scenario. The last category was the high carbon scenario, which in the future would not be affected by the epidemic, vigorous economic development, insufficient awareness of energy-saving development, and lack of implementation of relevant government policies, resulting in high emissions. Finally, the development projections of carbon emissions were obtained by combining the three trends of the seven factors [32].

#### 3.4.1. Population Scenario

**Figure 5:**
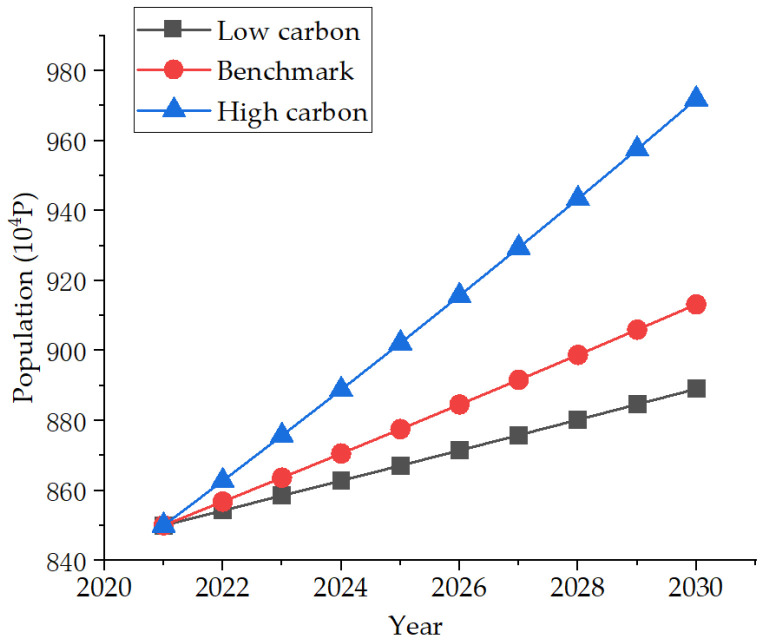
Three population development scenarios.

With the expansion of the city’s scale, a large number of people from the surrounding areas have poured into the city, resulting in an explosive growth of the population in the city. According to the bulletin of the seventh national census of Nanjing, the population growth rate in the past decade has been 16.38%, with an average annual growth rate of 1.53% [33]. However, with the development of society and the improvement of per capita education, people have a strong principle of autonomy in treating future generations, and more and more people are unwilling to have children. This has led to the decline of the birth rate and the slowdown of the growth of the working population, which is not conducive to the sustainable development of the city; the population growth rate was only 0.8% in 2020. Therefore, taking the 0.8% population growth rate as the benchmark scenario, The high carbon scenario is set as the government alleviates the employment pressure and marriage pressure on young people, and promotes a series of policies to make young people willing to have children and raise the next generation. For this, 1.5% is set as the development trend. Due to the increasing social pressure, the younger generation is more and more reluctant to have children because of their livelihood. This is set as a low-carbon scenario, with a growth rate of only 0.5%. The specific development trend is shown in Figure 5.

#### 3.4.2. Industrial Output Value Scenario

**Figure 6:**
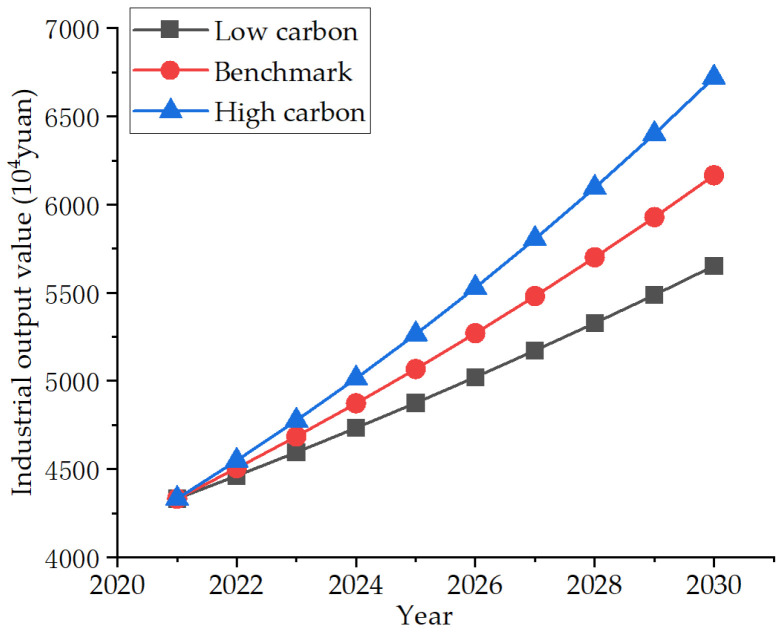
Three industrial output value development scenarios.

Since the 13th “five-year” plan [34], we have adhered to being innovation-driven, developed new innovative companies, and gradually moved the five traditional high energy-consuming industries of oil refining, chemical industry, cement, steel, and electric power away from the urban area and to the surrounding cities. Therefore, the proportion of industry and its secondary industry in the total urban GDP has gradually decreased, and the benchmark scenario is the average annual growth rate of the industrial output value of 4% in the recent five years. In the next few years, the industry will be transformed successfully—the traditional industries of oil refining, chemical industry, cement, steel, and electric power will be gradually replaced by technology-intensive industries such as new energy and new materials, and the industrial output value will be greatly increased. This is set as a high-carbon scenario with a growth rate of 5%. Due to the impact of the pandemic in recent years, small and medium-sized enterprises have closed down, personnel has been laid off, orders of some traditional industrial departments have decreased, performance has declined, and the total industrial output value has also decreased. This is set as a low-carbon scenario, with a growth rate of 3%. The specific development trend is shown in Figure 6.

#### 3.4.3. Industrial Labor Productivity Scenario

**Figure 7:**
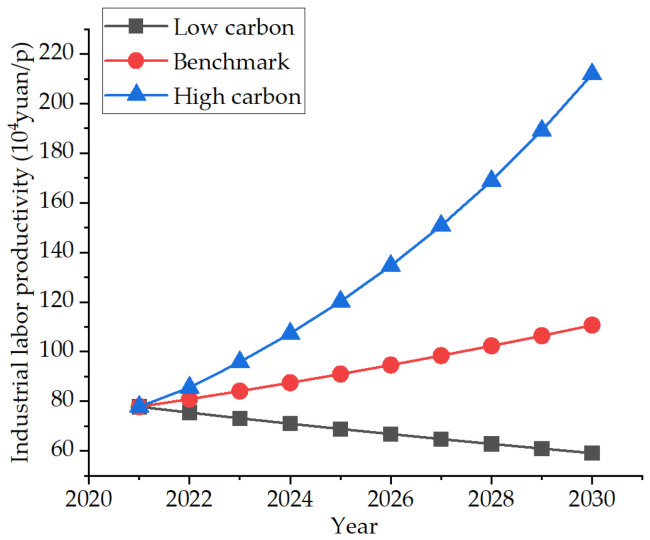
Three industrial labor productivity development scenarios.

Industrial labor productivity refers to the ratio of industrial output value to the industrial population. Since 2020, affected by the COVID-19, industrial labor productivity has experienced negative growth for the first time. COVID-19 has led to the shutdown of enterprises, the unemployment of personnel, and the decline of industrial output value. However, in the past decade, it has maintained high-speed growth, technological progress, and the improvement of the professionalism of employees. Assuming that the impact of COVID-19 would last for the next 10 years, which will eventually lead to the bankruptcy of small and medium-sized enterprises and the increase of unemployed people, the low-carbon scenario is set as −3% [35]. In the future, the pandemic will be well managed, the enterprises will return to work and the personnel will go to work. The high-carbon scenario is set as 10%. The pandemic always exists and occasionally breaks out. The development maintains a stable status quo. For this, a benchmark scenario is set, with a growth rate of 4% [36]. The specific development trend is shown in Figure 7.

#### 3.4.4. Industrialization Rate Scenario

**Figure 8:**
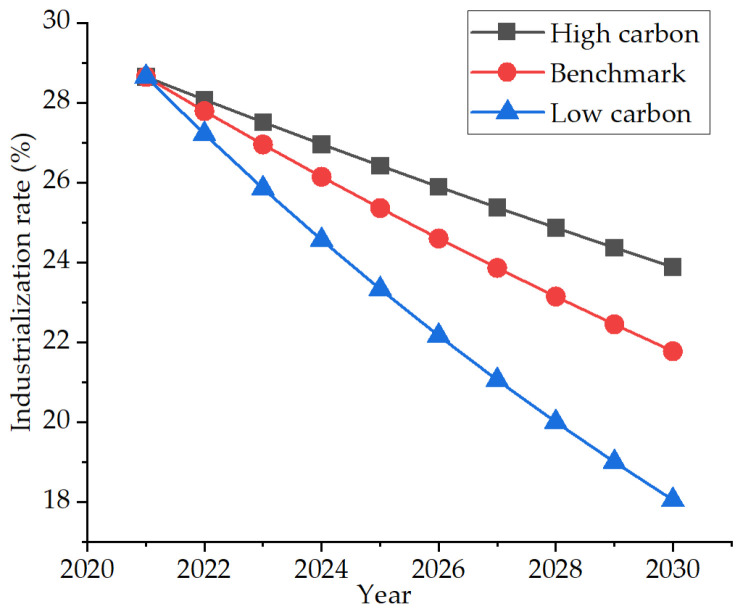
Three industrialization rate development scenarios.

The industrialization rate refers to the ratio of industrial GDP to regional GDP. After 2002, Nanjing adjusted its development strategy based on the actual needs of development, implementing the development strategy of “industry-first policy”, and the secondary industry began to further accelerate its development. However, since 2012, with the rise of the financial industry, tourism, and information service industry, the proportion of industry had gradually decreased, and the tertiary industry had gradually become the pillar industry of Nanjing. Therefore, it was predicted that the industrial output value would gradually decrease in the future. Therefore, based on the average annual growth rate of −3% in the past eight years, due to the slow industrial transformation and the timely adjustment of the industrial structure, it is set as the high-carbon scenario with a growth rate of −2%, and the low-carbon scenario is the rapid industrial transformation and the rise of the information service industry, which greatly reduces the industrialization rate with a growth rate of −4%. The specific development trend is shown in Figure 8.

#### 3.4.5. Energy Intensity Scenario

**Figure 9:**
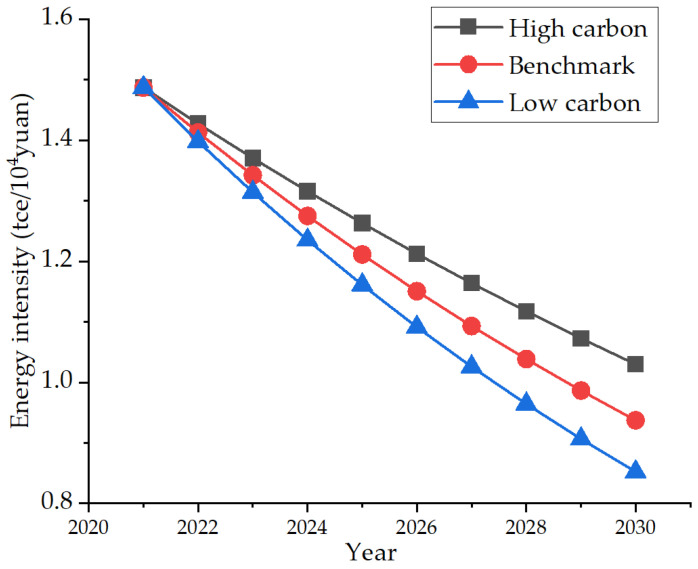
Three energy intensity development scenarios.

During the 12th Five-Year Plan period, Nanjing’s energy consumption per 10,000 yuan GDP decreased by 25.67% in total, with remarkable results in energy conservation and consumption reduction, and won the “outstanding contribution award to provincial energy conservation”. The city had invested nearly 13 billion yuan, implemented more than 550 key energy-saving technological transformation projects, closed down and transformed high-energy consuming industrial production processes and eliminated backward production capacity, and achieved positive results. Therefore, based on the average annual energy intensity growth rate of −5% during the 12th Five-Year Plan period, the high-carbon scenario is set because the industrial transformation results are not ideal, the industry continues to occupy a large proportion and energy consumption is still large. For this, the growth rate is set as −4%. The low-carbon scenario refers to the slow decline of the proportion of the secondary industry, the gradual growth of the proportion of the tertiary industry, the decline of the proportion of industrial output value, and the reduction of energy consumption. For this, the growth rate is set as −6%. The specific development trend is shown in Figure 9.

#### 3.4.6. R&D Intensity Scenario

**Figure 10:**
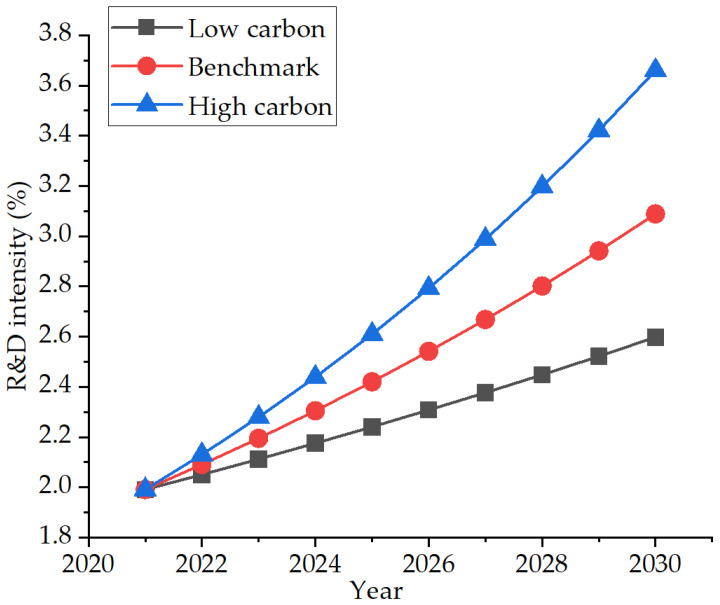
Three R&D intensity development scenarios.

During the 12th Five-Year Plan period, there were 27,123 effective invention patents in the city, a year-on-year increase of 30.88% [37]. The proportion of R&D investment in GDP, the number of invention patents owned by 10^4^ people, the number of talents in the national “thousand people plan”, the number of national science and technology platforms, and the number of awards for national scientific and technological achievements ranked first in the province for many consecutive years. By 2020, the R&D expenditure of industrial enterprises above designated size would account for 1.45% of the main business income, the R&D institutions of industrial enterprises above designated size would be fully covered, 100 enterprise technology centers recognized at or above the provincial level would be added, and 95 invention patents would be applied for by industrial enterprises per 10 billion yuan output value [38]. Comprehensively promoting the technological transformation of enterprises, and the investment in industrial-technological transformation accounts for 65% of the industrial investment. Therefore, based on the average annual growth rate of R&D intensity since the 12th Five-Year Plan, the low-carbon and high-carbon scenarios were set as 3% and 7%, respectively, according to the benchmark scenario. The specific development trend is shown in Figure 10.

#### 3.4.7. Energy Structure Scenario

**Figure 11:**
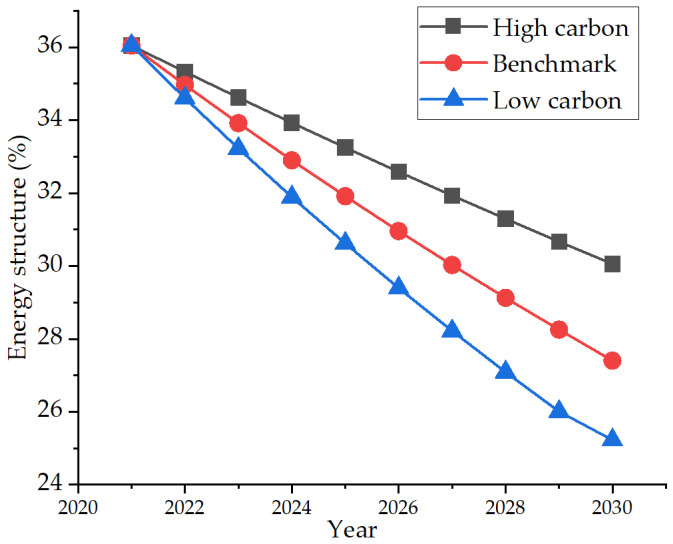
Three energy structure development scenarios.

In recent years, the proportion of petrochemical and steel in the total output value of industries above the designated size had decreased from 38.8% to 27.3%, down 11.5 percentage points; The proportion of electronic information and automobile industry increased from 24.2% to 37.6%, an increase of 13.4 percentage points [39]. The proportion of six categories of nine strategic emerging industries in the industrial economy exceeded the three traditional industries of petrochemical, iron, and steel, and building materials for the first time, which prompted nodal changes in the industrial structure, marking that the situation of using traditional energy to achieve economic development had changed. Therefore, based on the average annual growth rate of −2% in the recent ten years, the low-carbon scenario is set as that the proportion of emerging industries in Nanjing continues to increase, and the demand for traditional energy gradually decreases, with the growth rate set at −3%. The high carbon scenario is in the coming years. Due to technology and policy reasons, the development of emerging industries is restricted and slow, and its growth rate is set as −1%. The specific development trend is shown in Figure 11.

#### 3.4.8. CO_2_ Emissions Scenario

**Figure 12:**
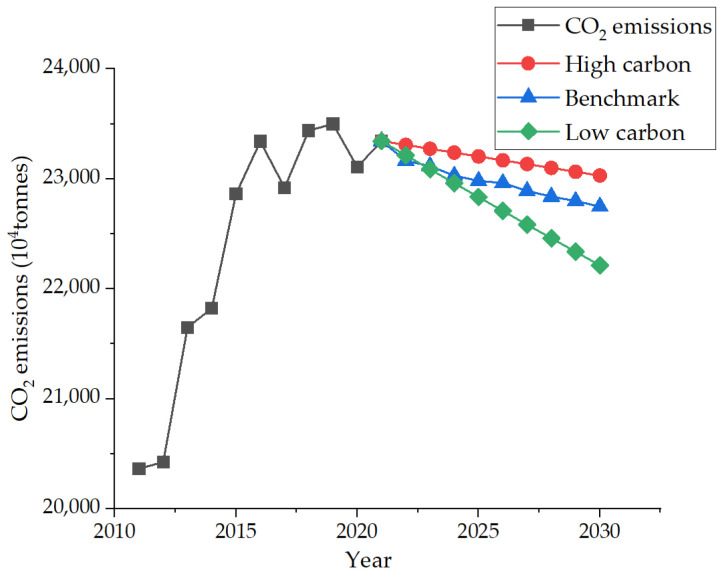
Three CO_2_ emissions development scenarios.

Based on the data obtained from the analysis of three scenarios of seven carbon emission factors, the development scenario of Nanjing under the action of seven factors is finally brought into the STIRPAT model. Finally, the average growth rates of carbon emissions under low-carbon, benchmark, and high-carbon scenarios are −2%, −1.5%, and −0.6%, respectively. It can be concluded that the industrial carbon emission of Nanjing in 2030 is 229.95 million tons under the high carbon scenario, 226.9165 million tons under the benchmark scenario, and 220.906 million tons under the low carbon development scenario. As shown in Figure 12, it can be seen that carbon emissions generally show a downward trend under the three development trends. The highest point was 235.02 million tons in 2019 before the outbreak of the pandemic. On the one hand, due to the outbreak of the pandemic in 2020, the overall economic development rate has decreased, and the power generation, industrial activities, and transportation have also decreased, thus reducing the carbon dioxide emissions caused by fossil energy. People’s lifestyles are also gradually changing. They study at home, work and travel less, which greatly reduces the energy demand. On the other hand, it shows that the comprehensive work plan for energy conservation and emission reduction in the 13th five-year plan [40] has achieved success by the end of 2019, including adjusting the industrial structure, reducing comprehensive energy consumption, optimizing the energy structure, etc., which has led to the reduction of industrial carbon emissions. 

### 3.5. Discussion on Carbon Economy 

Through the GRPM model and scenario analysis, the carbon emission is finally obtained. In the process of industrial production, the carbon emission is more linked to the economy, and the carbon released in each step of the production process is evaluated. At present, the carbon cost evaluation is mainly linked to money. In the production process, including material selection, processing, and finished products, there are direct or indirect carbon emissions, which can be solved by heuristic carbon balance [41]. Each industrial carbon emission process is divided into three stages: design, operation, and completion. Each stage corresponds to energy consumption in order to calculate carbon dioxide emission and estimate carbon emission cost. It can also be analyzed from the perspective of social cost. The so-called social cost is the economic and social consequences caused by carbon emissions. Calculating the cost of industrial carbon emissions can be linked to the cost of environmental maintenance [42]. However, due to the huge uncertainty in the economic evaluation of carbon emissions, determining a practical way to evaluate carbon value is more conducive to evaluating the actual economic impact caused by industrial carbon emissions from decision-makers. At present, there are two superior evaluation methods, one is through the joint probability density function [43], and the other is through meta-analysis [44]. This paper studies industrial carbon emissions to establish the main influencing factors of carbon emissions, infers a probability density function, and reduces the uncertainty of carbon evaluation by determining the relationship between variables.

## 4. Conclusions and Policy Suggestion

### 4.1. Main Conclusions

(1) Among the industrial carbon emissions in Nanjing, the overall industrial carbon emissions showed a high-speed growth trend from 2006 to 2015. In order to meet the requirements of the 13th Five-Year Plan, the overall industrial carbon emissions showed a low growth trend and even a negative growth from 2016 to 2020, indicating that Nanjing had made great achievements in these years and had the certain foundation for the development of low-carbon economy in the future.
(2) Among the seven driving factors which could affect the industrial carbon emissions in Nanjing, the degree of impact on the industrial carbon emissions in Nanjing from large to small was as follows industrial energy structure > total population > industrial output value > labor productivity > R&D intensity > industrialization rate > energy intensity. The total population, industrial output value, labor productivity, and industrial energy structure were positively correlated with the industrial carbon emissions in Nanjing. Every increase by 1% of each factor increased carbon emissions by 0.52%, 0.49%, 0.17% and 0.12%, respectively. The three influencing factors of industrialization rate, energy intensity, and R&D intensity were negatively correlated with the industrial carbon emissions in Nanjing. Every increase by 1% of each factor inhibited the increase of the industrial carbon emissions in Nanjing by 0.03%, 0.07%, and 0.02%, respectively.
(3) According to the seven factors which could affect industrial carbon emissions in Nanjing and the calculation results of the carbon emissions from 2006 to 2020, it was concluded that with the rapid development of high carbon, Nanjing’s carbon emissions would reach 229 million tons in 2030, 226 million tons under the benchmark scenario and 220 million tons under the condition of low-carbon development.

### 4.2. Policy Suggestion

(1) Optimize the energy structure and increase the level of energy usage. Maintain a 1:1 ratio between energy consumption and production value by adhering to technological innovation, increasing R&D expenditure, maintaining R&D investment of more than 2%, reducing industrial energy intensity, and realizing the relationship between energy consumption and output value. Reduce the share of primary energy usage (coal and oil) as well. Increase the share of natural gas in energy consumption while also promoting the development and use of sustainable energy sources such as solar and wind. For example, vigorously support the creation of new energy demonstration projects in urban construction and people’s livelihoods, such as solar energy and geothermal energy. It was because of this that things improved. Improve the photovoltaic and wind power generation’s use efficiency.
(2) To deal with the rapid growth of the urban resident population, a slew of urban management issues have arisen, including a lack of public services, a scarcity of supporting resources, and increasingly serious environmental pollution, resulting in rapid carbon dioxide growth. As a result, the Nanjing municipal government must not only rationally recognize the problem of resource allocation and construct a faultless public service system, but also increase environmental protection public awareness, advocate low-carbon living, and develop low-carbon consciousness.
(3) Streamline industrial upgrading by optimizing the industrial structure [45]. Encourage the conversion of energy-intensive industries like steel, petrochemicals, and chemicals to technology-intensive industries like new energy and new materials, eliminate backward production capacity industries, build a low-carbon industrial chain dominated by technological innovation, aggressively develop low-carbon competitive industries, and accelerate the technological iteration and industrial upgrading. Reduce the amount of industry in Nanjing’s economic development through time and raise the proportion of tertiary industries such as tourism and finance.

## Figures and Tables

**Table 1:** STIRPAT model coefficient.

Index	Variable	Unit
	Total Industrial Carbon Dioxide Emissions (E)	10^4^ Tons
Population	Total urban population (P)	10^4^ P
Wealth	Total industrial production value (V)	10^4^ yuan
	Labor productivity (R)	yuan/p
	Industrialization rate (L)	%
Technical	Industrial energy intensity (EI)	tce/10^4^ yuan
	Industrial R&D intensity (RD)	%
	Industrial energy structure (ES)	%

**Table 2:** Correlation analysis between industrial carbon emission and influencing factors.

	lnE	lnP	lnV	lnR	lnL	lnEI	lnRD	lnES
lnE	1							
lnP	0.941 **	1						
lnV	0.969 **	0.976 **	1					
lnR	0.895 **	0.884 **	0.952 **	1				
lnL	−0.828 **	−0.890 **	−0.926 **	−0.955 **	1			
lnEI	−0.926 **	−0.972 **	−0.989 **	−0.956 **	0.955 **	1		
lnRD	0.719 **	0.717 **	0.806 **	0.935 **	−0.914 **	−0.837 **	1	
lnES	0.596 *	0.520 *	0.454	0.275	−0.17	−0.395	0.092	1

Notes: ** At level 0.01 (two-tailed), the correlation was significant; * at level 0.05 (two-tailed), the correlation was significant.

**Table 3:** Collinearity analysis R^2^ analysis.

Model	R	R^2^	Adjust R^2^	Error
1	0.998	0.996	0.991	0.019

**Table 4:** The predicted value of industrial carbon emission in Nanjing from 2021 to 2030.

Year	2021	2022	2023	2024	2025
Carbon emissions	231.6	231.2	230.23	229.8	229.6
Year	2026	2027	2028	2029	2030
Carbon emissions	228.9	228.4	228.0	227.5	226.9

## Data Availability

The data provided in this study were from Nanjing statistical yearbook 2006–2020.

## Abbreviations

Abbreviations	Definition
IPAT	impact = population × affluence × technology
STIRPAT	Stochastic Impacts by Regression on Population, Affluence, and Technology
GM (1,1)	grey model with one variable and one first-order equation
GRPM (1,1)	grey rolling prediction model with one variable and one first-order equation
IDA	index decomposition analysis
SDA	structural decomposition analysis
LMDI	logarithmic mean divisor index
GDP	gross domestic product
R&D	research and development
P	population size
A	affluence
T	technology level
E	carbon emissions
V	industrial output value
R	labor productivity
L	industrialization rate
EI	energy intensity
RD	industrial research and development intensity
ES	energy consumption structure
*C_rt_*	carbon emissions caused by energy consumption in year t in region r
*E_irt_*	consumption of class i energy in year t in region r.
*T_i_*	conversion coefficient of class i energy standard coal
*F_i_*	carbon emission coefficient of class i energy
IPCC	intergovernmental Panel on Climate Change
R^2^	coefficients of determination
VIF	variance expansion factor
lnE	carbon emissions logarithm
lnP	population logarithm

## Appendix A

**Table A1:** Original data of influencing factors of industrial carbon emission in Nanjing from 2006 to 2020.

Year	CO_2_ Emission (10^4^ tCO_2_)	Population (10^4^ P)	Industrial Output Value (10^8^¥)	Industrial Labor Productivity (yuan/P)	Industrialization Rate (%)	Energy Intensity (tce/10^4^¥)	R&D Intensity (%)	Energy Structure (%)
2020	23,107.12	850.00	4331.59	720,251.08	29.23	1.66	1.81	36.60
2019	23,500.20	843.00	4215.77	739,608.77	30.05	1.85	1.73	35.56
2018	23,438.58	833.00	4055.14	629,582.36	31.63	1.91	1.49	35.24
2017	22,919.48	827.00	3853.39	599,656.08	32.89	1.94	1.52	37.78
2016	23,540.03	823.00	3581.72	481,155.29	34.10	2.15	1.15	36.55
2015	22,862.82	821.61	3395.26	432,958.43	34.93	2.20	1.12	37.35
2014	21,821.93	818.78	3119.12	386,795.63	34.83	2.27	1.09	39.81
2013	21,648.02	816.10	2997.63	376,066.99	36.56	2.36	1.04	39.60
2012	20,423.35	810.91	2748.46	344,807.43	37.62	2.39	1.04	43.03
2011	20,364.64	800.76	2390.51	306,044.04	38.37	2.73	0.94	41.60
2010	17,836.18	771.31	2005.21	248,816.23	38.58	2.93	0.87	37.86
2009	14,665.08	758.89	1640.53	223,535.90	38.27	3.21	0.95	31.09
2008	14,060.99	741.30	1532.20	215,924.46	39.70	3.23	0.81	32.75
2007	14,805.93	719.06	1412.22	238,188.56	43.01	3.67	0.87	32.83
2006	14,295.80	689.80	1181.94	210,047.98	42.61	4.12	1.02	34.37

**Table A2:** Ridge regression fitting results.

	B	SE (B)	Beta	B/SE (B)
lnP	0.486	0.252	0.148	1.926
lnV	0.166	0.031	0.356	5.322
lnR	0.117	0.036	0.255	3.237
lnL	−0.033	0.144	−0.019	−0.225
lnEI	−0.067	0.046	−0.087	−1.458
lnRD	−0.017	0.074	−0.021	−0.221
lnES	0.519	0.179	0.229	2.902
Constant	2.144	1.644	0.000	1.304

**Table A3:** Conversion coefficient and carbon emission coefficient of standard coal.

Type	Conversion Coefficient	Carbon Emission Coefficient of Standard Coal
Raw coal	0.7143 (tce/t)	0.7476 (t/tce)
Coke	0.9714 (tce/t)	0.1128 (t/tce)
Crude oil	1.4286 (tce/t)	0.5854 (t/tce)
Gasoline	1.4714 (tce/t)	0.5532 (t/tce)
Kerosence	1.4714 (tce/t)	0.3416 (t/tce)
Diesel	1.4571 (tce/t)	0.5913 (t/tce)
Fuel oil	1.4286 (tce/t)	0.6176 (t/tce)
Nature gas	13.30 (tce/104 m^3^)	0.4479 (t/tce)

**Table A4:** Relative error table of fitting value of STIRPAT model.

Year	Actual Value	Fitting Value	Error
2020	23,107.12	24,313.56	0.0522
2019	23,500.20	23,649.88	0.0064
2018	23,438.58	22,791.13	−0.0276
2017	22,919.48	23,152.51	0.0102
2016	23,540.03	21,786.19	−0.0745
2015	22,862.82	21,510.69	−0.0591
2014	21,821.93	21,571.10	−0.0115
2013	21,648.02	21,198.35	−0.0208
2012	20,423.35	21,488.30	0.0521
2011	20,364.64	20,063.44	−0.0148
2010	17,836.18	17,723.61	−0.0063
2009	14,665.08	15,059.14	0.0269
2008	14,060.99	15,073.15	0.0720
2007	14,805.93	14,659.53	−0.0099
2006	14,295.80	13,933.71	−0.0253

**Table A5:** Prediction error table of GRPM model.

Year	Raw Data x^(0)^	Modeling Data y^(0)^	Prediction Data y^(0)^	Error Δ/%
2006	14,295.80	19,952.68		
2007	14,805.93	20,356.74		
2008	14,060.99	20,783.72		
2009	14,665.08	21,343.95		
2010	17,836.18	21,951.12		
2011	20,364.64	22,362.62	22,935.53	2.5619
2012	20,423.35	22,584.61	23,168.01	2.5832
2013	21,648.02	22,854.77	23,273.15	1.8306
2014	21,821.93	23,027.17	23,316.08	1.2547
2015	22,862.82	23,228.04	23,449.73	0.9544
2016	23,540.03	23,540.03	23,534.28	1.0008
2017	22,919.48	23,241.34	23,437.35	0.8434
2018	23,438.58	23,348.63	23,422.77	0.3175
2019	23,500.20	23,303.66	23,343.16	0.1695
2020	23,107.12	23,107.12	23,165.06	0.2507
